# An Evaluation of a Commercialized mHealth Intervention to Promote Physical Activity in the Workplace

**DOI:** 10.3389/fpubh.2022.740350

**Published:** 2022-03-15

**Authors:** Katie M. Di Sebastiano, Erica Y. Lau, Lira Yun, Guy Faulkner

**Affiliations:** ^1^Department of Sport and Exercise Sciences, Durham University, Durham, United Kingdom; ^2^Population and Physical Activity Laboratory, School of Kinesiology, University of British Columbia, Vancouver, BC, Canada; ^3^Vancouver Costal Health Research Centre, Centre for Clinical Epidemiology and Evaluation, Vancouver, BC, Canada; ^4^Department of Emergency Medicine, University of British Columbia, Vancouver, BC, Canada; ^5^Faculty of Kinesiology, Sport and Recreation, University of Alberta, Edmonton, AB, Canada

**Keywords:** mHealth, physical inactivity, sedentary behavior, scaling up, RE-AIM, implementation

## Abstract

**Background:**

UPnGO with ParticipACTION (UPnGO) was a commercialized 12-month workplace physical activity intervention, aimed at encouraging employees to sit less and move more at work. Its design took advantage of the ubiquitous nature of mobile fitness trackers and aimed to be implemented in any office-based workplace in Canada. The program was available at cost from June 2017 to April 2020. The objectives of this study are to evaluate the program and identify key lessons from the commercialization of UPnGO.

**Methods:**

Using a quasi-experimental design over 3 time points: baseline, 6 months, 12 months, five evaluation indicators were measured as guided by the RE-AIM framework. *Reach* was defined as the number and percentage of employees who registered for UPnGO and the number and percentage of sedentary participants registered. *Effectiveness* was assessed through average daily step count. *Adoption* was determined by workplace champion and senior leadership responses to the off-platform survey. *Implementation* was assessed as the percentage of participants who engaged with specific program elements at the 3-evaluation time points. *Maintenance* was assessed by the number of companies who renewed their contracts for UPnGO.

**Results:**

*Reach* across 17 organizations, 1980 employees participated in UPnGO, with 27% of participants identified as sedentary at baseline. *Effectiveness* Daily step count declined from 7,116 ± 3,558 steps at baseline to 6,969 ± 6,702 (*p* = <0.001) at 12 months. *Adoption* Workplace champion and senior leadership engagement declined from 189 to 21 and 106 to 5 from baseline to 12 months, respectively. *Maintenance* Two companies renewed their contracts beyond the first year.

**Conclusions:**

The commercialization of UPnGO was an ambitious initiative that met with limited success; however, some key lessons can be generated from the attempt. The workplace remains an important environment for PA interventions but effective mHealth PA programs may be difficult to implement and sustain long-term.

## Introduction

Being physically inactive, defined as not meeting physical activity (PA) guidelines (1), is a major risk factor for cardiovascular diseases, certain cancers, chronic respiratory diseases and type 2 diabetes (2). However, only 18% of Canadian adults are active enough to meet PA guidelines (3).

Increases in physical inactivity may be related to changes to the nature of work throughout the twentieth and twenty-first century. In the 1970s, 2 in 10 Americans had sedentary jobs; however, by the 2000s, this number had increased to >4 in 10 adults (4). With workers spending ~8.5 h per day working (5), the workplace, and particularly the office-based workplace, is an important setting to promote PA. Mobile health (mHealth) interventions may be an effective way to promote PA in the workplace. A systematic review of 25 experimental and quasi-experimental studies of mHealth interventions in the workplace found that 56% of studies reported a significant increase in PA (6). However, workplace PA interventions have demonstrated low-quality evidence and limited effectiveness (7, 8) with the effectiveness highly dependent not only on the type and content of the program, but the population, study characteristics, and methodology as well (7). Additionally, there is a paucity of studies examining the dissemination of effective interventions in real-world settings.

To address changes in these health behaviors, ParticipACTION, a Canadian non-profit which promotes PA nationally, developed a mHealth intervention named UPnGO with ParticipACTION (UPnGO). The intervention was designed to take advantage of the ubiquitous nature of mobile fitness trackers and mHealth applications so that it could be delivered at a population level to any workplace in Canada. UPnGO began as a 6-week workplace PA initiative aiming to increase habitual PA (steps) during the workday. Higher step counts have been associated with lower risk of all-cause mortality, and lower risk of cardiovascular morbidity and mortality (9). Intervention components included self-monitoring of steps and action planning behaviors via a web/mobile app with incentives and organizational support, including role modeling from senior management and program endorsement. A significant increase of ~540 steps/day at week 6 was found among participants in workplaces with optimal levels of implementation, defined as high fidelity (supportive environment for PA) and dose-received (participant program engagement) (10).

With the initial success of the UPnGO program, ParticipACTION then scaled-up the program. Scaling-up is defined as deliberate efforts to increase the impact of successfully tested health interventions to benefit more people and to foster policy and program development on a sustainable and lasting basis (11). There is a growing desire to scale-up evidence based interventions in various settings; however, this process is notoriously difficult to achieve with the majority of interventions in lower- and middle-income as well as high income countries facing significant barriers and pitfalls to their success (12). As UPnGO is a workplace-specific intervention, commercialization—scaling-up through entering into partnerships with private entities for financial reimbursement (11), was determined to be the most applicable approach. During the scale-up phase, workplaces could access the UPnGO program at cost.

This manuscript describes the changes made to UPnGO with ParticipACTION for the purpose of commercialization. The specific objectives are to (1) evaluate the commercialized UPnGO program using the RE-AIM framework; and (2) identify key lessons to inform future considerations in the commercialization of PA programs.

## Methods

### Scaling-Up UPnGO With ParticipACTION—Intervention Description

The commercialized UPnGO program was a 12-month workplace PA intervention that evolved from the initial piloted 6-week intervention (10). Development of the program was guided by the Behavior Change Wheel (BCW) model (13) and grounded in the concept that increasing employees' behavior change capability/skills (e.g., action planning, self-monitoring) and creating an active workplace culture (i.e., a supportive social environment as reflected by leadership support of PA and PA opportunities) would increase employees' engagement in PA (i.e., daily steps) and decrease sitting behaviors at the workplace. A complete description of the program development and theoretical underpinnings can be found in Lau and Faulkner (10).

The initial UPnGO pilot phase study was a 6-week program, which consisted of three essential elements across two levels of intervention (individual and organizational/environmental): (1) education and training on PA behavior change skills (individual); (2) real-world PA opportunities (organizational); and (3) leadership support of engagement in the UPnGO program and PA participation in the workplace (organizational). These elements were delivered both on- and off-platform the UPnGO web/mobile app. The on-platform program component includes an UPnGO web-based and mobile application that could be synched with wearable PA trackers (e.g., FitBit). The off-platform components included weekly themes, walking activities and opportunities for participants to interact within the workplace. Workplace champions supported both on- and off-platform activities.

The decision to extend the time-line of the program from 6 weeks to 1 year was based on feedback that Canadian companies would be less interested in paying for a short-term program. The 12-month program followed the same principles as the 6-week program, however overall monthly themes were added to the program with supporting weekly themes where appropriate. A 1-month long challenge was included every quarter (4/year), such as *Reboot your Commute* which challenged participants to engage in PA during their commute. Additionally, UPnGO participants were no longer provided devices to monitor PA as in the pilot program and were expected to use their own device (wearable technology or phone) to monitor activity. Participants who did not have access to these devices were able to manually record PA, but not their daily step count through the web-based platform. A summary of the differences between the 6-week and 12-month program can be found in Table 1.

**Table 1:** Scaling-up UPnGO.

**Program feature**	**6-week program**	**12-month program**
**Individual level components**		
Education and training on PA behavior change skills	•Web-based and mobile platform •Weekly themes and challenges	•Web-based and mobile platform •Monthly themes with supporting weekly themes •1 Challenge per quarter (4/year)
Self-monitoring	•Web-based and mobile platform •Wearable fitness device provided	•Web-based and mobile platform
**Organizational level components**		
Real-world opportunities for PA	•Weekly walking activities •Workplace-based opportunities for PA	•Walking activities at the discretion of the champions •Workplace based opportunities for PA at the discretion of the champions
Leadership support of UPnGO and participating in PA in the workplace	•Formal communication of support •Senior management to participate in at least 50% of events •Permission to participate (flexible work schedule)	•Formal communication of support •Senior management to participate in at least 50% of events •Permission to participate (flexible work schedule)
Company characteristics	•Ontario and British Columbia only •Small to medium (1–499 employees) size companies •Employees present in office most days •Desk-centric office environment •No-existing workplace PA intervention	•All of Canada •No size limits •Employees present in office most days •Desk-centric office environment
Implementation	•Weekly check-in with implementation specialist •See Lau and Faulkner (10) for details	•Monthly check-in with implementation specialist •6- and 12-month reports detailing outcomes with recommendations to improve implementation

#### Individual Level: Education on PA Behavior Change Skills and Self-Monitoring

The individual-level components aimed to enhance an individual's capability in self-regulating their PA behaviors. This was primarily delivered through the on-platform component of the UPnGO program. It included a web-based and mobile application that contained (1) educational content, delivered via blogs, notifications, stream posts, and videos, related to behavior change skills, including goal-setting, action planning, barrier identification/problem solving, and self-monitoring; (2) a platform for self-monitoring (tracking) steps by using a wearable device; and (3) a platform for self-monitoring activities related to action planning, such as planning a walking meeting, or setting standing reminders. The application also provided participants with a set of interactive tools to increase program engagement. This included emails, prompts and notifications to support individuals in achieving their PA goals and an incentive system to promote and sustain program engagement throughout the intervention. There was also a stream post function for individuals to seek and provide peer support, a group/company calendar for creating and promoting social PA events, and a leaderboard that ranked participant activity levels in order to foster positive inter- and intra-departmental competitions.

#### Organizational Level: Leadership Support and Opportunities for PA

The organizational-level components aimed to provide organizational support to make PA a socially acceptable choice. These activities were mostly operated outside the UPnGO platform. Leadership support was reflected by three aspects of UPnGO: communication of support, role modeling, and permission. Senior leadership at each participating worksite was expected to provide a formal communication of support to their employees, such as through company-wide email/official announcements about UPnGO. Senior management was expected to serve as a role model to their employees by actively engaging in at least 50% of the UPnGO events. They were also recommended to work closely with their managers on every monthly theme to ensure that employees were allowed to have a flexible work schedule (i.e., permission and leadership endorsement) to engage in the UPnGO activities.

Monthly real-world PA activities were organized at the worksite by UPnGO workplace champions and related to the monthly theme. Examples of the most popular monthly themes included *Action Plan It*, focusing on developing action planning skills, *Unite to Ignite*, focusing on increasing social support for PA, and *Pump It Up*, aiming at increasing physical activity intensity.

Optional organizational-level components included environmental prompts (e.g., signage) and engaging organizational champions to organize weekly PA opportunities and program emails.

### Participants

A similar sampling frame was used for the commercialization of UPnGO as with the pilot phase of the study as described by Lau and Faulkner (10). Companies were identified through ParticipACTION's personal networks, as well as companies that publically declared a focus on encouraging physically active workplaces and workplace wellness, and companies recognized as “top employers” and “top places to work” were targeted. However, there were a couple of key differences including eligibility for companies across Canada (not limited to Ontario and British Columbia) and there were no limits set on company size. Company recruitment occurred through targeted recruitment strategies by the implementation specialist, with initial contact occurring primarily through ParticipACTION's presence at various industry conferences. Cost for UPnGO varied based on company size, with larger companies receiving pro-rated program rates. The maximum cost for the program was $100 CAD per participant.

A total of 17 companies enrolled in the commercialized UPnGO program from June 2017 until April 2019. Similar to the pilot, the characteristics of the companies varied in terms of organization size. This included 11 companies with <100 employees, 3 companies with 101–200 employees, 2 companies with 201–300 employees, and 3 companies with >301 employees. The companies also covered various sectors including public administration (5), financial (4), management (3), professional, scientific and technical services (2), agriculture (1), energy (1), and telecommunications (1). Employee eligibility was determined by the individual company as the funding model required employers to pay per participant.

### Implementation Procedures

Program implementation proceeded as described by Lau and Faulkner (10). In brief, the implementation team worked with the workplace contact to identify and recruit executive and program champions. These champions then received training and materials as to how to recruit participants, after which a program launch event was held to encourage participants to download the app and register for the program. The launch event was promoted through usual workplace communications (e.g., emails; newsletters; posters). Modifications to implementation procedures were made to account for the extended timeline. These included monthly emails regarding themes from the implementation specialist (instead of weekly), quarterly meetings with workplace champions to review progress, and companies were provided with 6- and 12-month reports summarizing the program outcomes with recommendations to support and improve program implementation. These reports were generated based on the PA data extracted from the app/web-based platform as well as an off-platform survey. This survey included markers of PA and sedentary behavior through the International Physical Activity Questionnaire—Short Form (14) and Occupational Physical Activity and Sedentary Behavior Questionnaire (15).

### Scaling-Up UPnGO Evaluation With the RE-AIM Framework

UPnGO with ParticipACTION was launched in April 2017 and was concluded in April 2020. To evaluate the program, a quasi-experimental design with no control group was employed. Three time points were included in the evaluation: program initiation (baseline), 6 months post-baseline (6 months), and 12 months post-baseline (12 months). The RE-AIM framework was selected to guide the evaluation of the commercialized UPnGO program (16). The framework provides 5 dimensions that relate to health behavior interventions and can improve sustainable implementation of interventions. These include Reach, the number, proportion, and representativeness of individuals willing to participate; Effectiveness, the impact of the intervention; Adoption, the number, proportion, and representativeness of intervention agents who are willing to initiate the program; Implementation, fidelity to the elements of the intervention's key function and components; and Maintenance, the extent to which the program becomes institutionalized. Glasgow et al. (16) provides a detailed description of the RE-AIM framework. Data collection and evaluation activities were ongoing during the 3 years of the program, with a formal annual report reflecting these RE-AIM metrics occurring in April of each year of the program.

#### Measures

Reach was assessed by two primary metrics: number and percentage of employees who registered for UPnGO at registered organizations, and the number and percentage of physically inactive participants registered. The number and size of the companies who registered for UPnGO influenced these metrics significantly. However, taken together, these metrics assess the various levels of reach required for the successful commercialization of an intervention: organizational, individual, and targeted population. As physically inactive office workers were the target demographic for the UPnGO program; 3 separate metrics were used to determine the successful recruitment of inactive participants at baseline; participants (1) with a step count <5,000 steps per day at baseline (17), based on the step count extracted from the synced activity monitor; (2) self-reported <150 min MVPA per week at baseline, calculated from the baseline IPAQ questionnaire (14); and (3) self-reported sitting for >6 h per day, derived from the occupational PA and sedentary behavior questionnaire (15) in the off platform survey.

Effectiveness was assessed through average daily step count as the program aimed to increase physical activity (as measured by steps). Participants' de-identified daily step counts were automatically synched to the UPnGO platform through their personal activity tracking devices (e.g., Garmin, Fitbit, Jawbone, Google fit app, iOS Health app etc.). As such, each individual's device is subject to its own internal logic for calculating steps; however, for the purpose of our analysis all recorded steps through devices were considered the same. A valid day was defined as any day with step counts between 1,000 and 40,000 (18). Average time point step counts (baseline, 6 months, 12 months) were determined by averaging the daily step count over the first 2 weeks of the month. To maximize the number of users with available step data, users must have had a daily step count for at least 5 days over 2 weeks at each time point to be included in the analysis. Users were not able to manually record their step count however, they were able to self-report other types of PA as well as health habits on the platform. This encouraged engagement for users who did not own a step-tracking device.

All analyses were conducted using SPSS version 26.0. Descriptive characteristics were extracted from the implementation specialist records and PA metrics (steps and activity tracking) were extracted from the on-platform database where applicable. Of the 1,980 registered users, 667 (33.7%) had valid step data at baseline, 517 (26.1%) had valid step data at 6-months and 193 (9.7%) had valid step data at 12 months. As such, we used a conservative intent-to-treat (ITT) approach, whereby participants are only required to have valid step data at baseline to be included in the analysis. For participants who did not provide complete step data at later intervention time points (6 and 12 months), we conservatively assumed no change in outcomes by carrying forward baseline values. We also performed a sub-analysis on users who had complete step count data (all 3 time points). The step count data is extracted from the online platform, completely de-identified, as such it is not possible to assess whether the demographic characteristics of users with step count data differ from users without step count data. One-way repeated measures ANOVA were used to assess differences between the 3 time points. Changes in step count over the 3 time points was also compared between active and inactive users at baseline using mixed two-way repeated measures ANOVAs and reported using simple main effects with Bonferroni correction for multiple comparisons to assess interactions between the groups. Statistical significance was set at *p* < 0.05.

Adoption was determined by two metrics: the number of workplaces who were willing to implement the program, and workplace champion and senior leadership responses to the off-platform survey to assess the number of agents willing to implement the program (change agents). The pilot program of UPnGO revealed that engagement of senior leadership and workplace champions were integral to success by creating a workplace that supports PA (10). We tracked the number of users who self-identified as either a workplace champion or in a senior leadership position within the company on the off-platform survey. Implementation was assessed as the percentage of participants who used specific program elements at the 3-evaluation time points. These included logging into the UPnGO platform, tracking their steps, tracking their healthy habits, and tracking their activity. This metric identifies individual level use of the program. Maintenance was assessed by the number of companies who renewed their contracts for UPnGO beyond their initial 1-year commitment to assess the institutionalization of the program. An overview of key indicators is presented in Table 2.

**Table 2:** RE-AIM framework evaluation.

**Component**	**Indicator**	**Outcome**			
Reach	# and % of employees reached	1,980 (29% of 6,913 employees)			
	# and % of sedentary participants: a) <5,000 steps/day at baseline b) <150 min MVPA/week at baseline (self-report) c) Sit >6 h/day (self-report)	a) 170 (27% of 629 participants with step data at baseline) b) 406 (35.7% of 1,135 survey respondents) c) 923 (82.2% of 1,123 survey respondents)			
Effectiveness	Physical activity: step count (mean ± SD)		**Baseline**	**6 Months**	**12 Months**
		ITT (*n* = 667)	7,116 ± 3,558	6,642 ± 6,382^*^	6,969 ± 6,702^*^^+^
		Participants with data at all timepoints (*n* = 130)	7,915 ± 3,656	7,622 ± 3,312	6,905 ± 3,531^*^^+^
Adoption	# of organizations	17			
	Change agents adopting UpnGO		**Baseline**	**6 Months**	**12 Months**
	# of survey responses	Champions	189 (recruited)	32	21
		Senior leadership	106	16	5
Implementation	Overall program component use % of registered participants who engaged with specific program elements		**Baseline**	**6 Months**	**12 Months**
		Logged-in	98%	47%	30%
		Tracked steps	42%	34%	25%
		Tracked healthy habits	39%	10%	7%
		Tracked activities	55%	39%	28%
Maintenance	# and % of organization who renewed their contract beyond the 1st year	2 (12% of 17 organizations)			

* *Represents significant difference from baseline.*

+ *Represents significant difference from 6 months*.

## Results

### Reach

A total of 17 organizations with 6,913 employees were eligible for at least 1 year of UPnGO. Of the employees, 1980 (29%) registered for the program. At baseline, 27% of participants recorded <5,000 steps per day, while 36% of survey respondents reported that they performed <150 min of MVPA/week, and 82% of respondents reported sitting for >6 h/day.

### Effectiveness

UPnGO aimed to increase daily step count, however, there was a modest decline in step count between baseline and 6 months in the ITT analyses (Baseline: 7,116 ± 6,684 steps/day vs. 6 Months: 6,642 ± 6,382 steps/day, *p* < 0.001). By 12 months there appears to be a rebound in steps (12 Months: 6,969 ± 6,702 steps/day; vs. 6 months, *p* < 0.001); though this number is still significantly lower than baseline (vs. baseline *p* = 0.010). When we looked at only the participants who had step data at all 3 time points, we observed a decline in step count at 12 months (Baseline: 7,915 ± 3,656 steps/day, 6 Months: 7,622 ± 3,312 steps/day, 12 Months: 6,905 ± 3,531 steps/day; *p* < 0.001). When we compared inactive vs. active participants at baseline over the 3 time points, we observed no significant change in the step count of the inactive participants (<5,000 steps at baseline) (Baseline: 4,700 ± 1,812 steps/day, 6 Months: 4,798 ± 2,283 steps/day, 12 Months: 4,742 ± 2,680 steps/day; *p* = 1.000). However, active users appear to be driving the observed rebound in step count from baseline to 12 months (Baseline: 10,476 ± 2,506 steps/day, 6 Months: 9,207 ± 3,062 steps/day, 12 Months: 10,066 ± 3,511 steps/day; *p* < 0.001).

### Adoption

A total of 189 workplace champions were recruited across the 17 companies; however, at 12 months only 21 responded to our survey. Similarly, 106 senior leaders responded to the baseline survey. This number dwindled to 5 by 12 months suggesting declining engagement in program assessment after initial adoption by senior management.

### Implementation

Participants' use of all program components declined over the 12 months. This included system logons (baseline: 98%; 6 months: 47%; 12 months: 30%), step tracking (baseline: 42%; 6 months: 34%; 12 months: 25%), activity tracking (baseline: 55%; 6 months: 39%; 12 months: 28%), and healthy habit tracking (baseline: 39%; 6 months: 10%; 12 months: 7%).

### Maintenance

Only 2 (12%) out of the 17 companies renewed their contract, both of these companies were recruited in the first year of UPnGO and renewed their contracts for both year 2 and 3 of UPnGO. This suggests limited maintenance of the UPnGO program based on our criteria. We are unable to present results on the maintenance of the UPnGO program at the individual level as step data was not collected following the 1-year program.

## Discussion

The intent of the commercialization of UPnGO was to create a sustainable and financially viable workplace intervention. The commercialized UPnGO program demonstrated limited success. A total of 17 organizations with almost 7,000 employees purchased the commercialized UPnGO program suggesting organizational interest in such programs. However, only ~30% of those employees registered for UPnGO and even fewer of those registered users were considered inactive at baseline, based on the study metrics. Program engagement significantly and steadily declined over the 1-year program, with limited effectiveness, adoption, and implementation of the UPnGO program. Only two organizations renewed their UPnGO contracts beyond the first year, suggesting a lack of sustainability of the program. UPnGO with ParticipACTION has now been retired and key learnings from the program are being adopted into future ParticipACTION initiatives. UPnGO's commercialization process may provide significant insight into some of the challenges in implementing a national workplace physical activity mHealth intervention.

### Declining Program Engagement

Sufficient program engagement is necessary for any digital health intervention to have an effect (19). Declining engagement with UPnGO may have had a significant impact on program sustainability. In a recent meta-analysis, a small but statistically significant positive association between digital health engagement (based on all usage measures) and PA was reported (20). Of the 1,980 employees who registered for UPnGO, only 193 participants (~10% of total participants) had valid step data at 12 months and the number of users who logged on to the UPnGO system declined from 98% of participants at baseline to 30% of participants at 12 months. This lack of engagement with the UPnGO program may contribute to the decline in step count observed in the current study; and prolonged engagement likely remains a challenge to sustain PA mHealth interventions.

However, declining engagement with long-term mHealth interventions is not unprecedented. Hermsen et al. (21) demonstrated exponential decay in Fitbit use, with ~74% of study participants using their Fitbit after 100 days and only ~16% of participants using their Fitbit at 320 days. In a study of long-term PA app use, all of the participants (100%) had stopped using the app by 10 months (22). Three general usage patterns for PA apps and wearable devices have been identified, including short-term use, long and consistent use, and intermittent use (23), though more specific usage patterns have been identified (24, 25). Considering these features of mHealth initiative, re-engagement of intermittent users and strategies to promoted continued use should be a key feature of future workplace mHealth interventions.

Associated with declining engagement, the 6-week pilot study Lau and Faulkner (10) revealed that low implementation of UPnGO resulted in no significant changes in step count. In their study, implementation was assessed via a composite score of senior management role modeling, individual self-monitors, and self-monitoring of action planning activities. In the current study, at baseline, only 42% of users tracked their steps and 39% tracked their habits. These data suggest low levels of implementation at baseline which declined further over time. As such, it is unsurprising that PA did not increase. Additionally, there appears to be limited role modeling that occurred by 6 months as only 32 workplace champions and 16 individuals in senior leadership responded to the off-platform survey.

### Recruiting the Target Population

Like many PA interventions, UPnGO failed to recruit participants who would benefit most for the program—inactive adults. Inactive adults or participants who took <5,000 steps at baseline (17) accounted for only 27% of the users with available step data as baseline. This indicates that the recruitment procedures used in the UPnGO program were ineffective at reaching the target population. UPnGO used passive recruitment strategies including companywide emails and posters to alert potential participants and then included a site-specific launch event to encourage program registration. Additionally, there were no exclusion criteria to participate in the UPnGO program as UPnGO was available to all employees. Therefore, it is possible that the program may have been more appealing to active individuals and inactive individuals may simply not have been interested in a workplace PA intervention.

Recruitment and training were provided to workplace champions to help them recruit inactive participants in their worksite. Due to the scaling-up design we have no way of determining what additional recruitment strategies were employed at each site. Little is known about the most effective recruitment strategies for PA and specifically walking interventions. A systematic review of 47 studies examining recruitment in walking interventions identified the most effective recruitment strategies to be time and resource intensive, and require skilled research and recruitment staff (26). Therefore, it is unlikely that the training received by the UPnGO workplace champions was sufficient for effective recruitment of inactive individuals.

### The Reality of Commercialization

The nature of the UPnGO program lent itself to commercialization as it was designed to be implemented in office-based workplaces by companies that may have limited capacity to provide access or resources for PA promotion. This study demonstrated the feasibility of commercializing a workplace PA intervention with 17 companies participating. The 6-week pilot program was adapted to a product that consumers would want to purchase at a price point that was appealing and that was feasible for ParticipACTION to deliver. Several key features were changed to accomplish this goal: (1) the timeline was lengthened to 12 months, (2) participants were no longer provided with devices to track their steps and were asked to use their own device (wearable trackers or phone apps), (3) the longer timeline resulted in less frequent contact between the implementation specialists and the workplace champions and the champions had more autonomy within the UPnGO program. These changes resulted in a different UPnGO program delivered to participants in the 1-year program compared to the 6-week program, so much so that it rendered the real-world trial included in Lau and Faulkner's (10) hybrid evaluation irrelevant.

Commercializing UPnGO led to unforeseen pressures on ParticipACTION compared to the 6-week pilot program which may have hindered its success. This includes the expectations of a company paying for a commercialized PA program. Companies looking to purchase a workplace wellness program may not have the same level of commitment/expectation to supporting the implementation of the program. As such, there may be a perception of “Isn't that what we are paying you to do?” instead of an organizational internalization of the program principles. Additionally, as a non-profit, ParticipACTION did not have the resources to devote to the level of support required for company success to the extent required with the new model. The intent of the entire scaling-up process was to create a sustainable, income generating program that would increase PA in participants with minimal support from ParticipACTION.

In a commentary by Holtermann et al. (27), the authors identify some reasons why workplace interventions commonly fail, including a participatory approach to implementation, whereby human resources or the health and safety departments are responsible for the programs which are often limited financially. These programs are often not well-integrated into company processes (27). Additionally, they tend to focus on the health outcomes of individual participants and do not monitor the return on investment for the company (27), a barrier cited by employers to financially support ongoing programs (28). It is likely that UPnGO met with these pitfalls in many of the companies in which it was implemented, despite ParticipACTION's best efforts to address these issues.

As stated by Lau and Faulkner (10), UPnGO had an extremely short timeline for development, from initial program development to the hybrid testing which influenced the program design. These time pressures were still present in the scaling-up of the UPnGO program. This is not an uncommon concern when scaling-up interventions. In their framework describing the pathways for scaling-up public health interventions, Indig et al. (29) notes that both funding and policy pressures may result in skipping one or more of the 4 steps in the scaling-up framework. These steps include theoretically-based study design, an efficacy trial, effectiveness trials, and program dissemination. These pressures can limit the capability to revise programs when red flags (e.g., evidence of declining engagement) are identified in the scaling-up process.

### Program Characteristics

It is possible to increase PA long-term (>12 months) through behavior changes interventions. A systemic review of 9 RCTs by Wahlich et al. (30) demonstrated significant increases in both steps and moderate to vigorous PA at both 1- and 4-years post study intervention. However, the interventions included in this meta-analysis were short-term (generally no longer than 12 weeks) interventions. This suggests that an alternative approach, repeated short-term (<12 week) challenges, may have been more effective in increasing PA in UPnGO. Specifically, repeated short-term interventions or workplace challenges that target specific behaviors throughout the year may be more effective at long-term behavior change than the long-term intervention used in the current model. The program changes, as summarized in Table 1, including the increased program length and reduced contact with the implementation specialist, also resulted in declining levels of implementation during the 12 months of the programs, which may also have impacted the effectiveness of the UPnGO intervention and may be addressed through repeated short-term challenges.

In addition to program changes for commercialization, variability in company size may have also significantly impacted the success of UPnGO. The smallest company had just 22 employees while the largest had over 4,000. In an evaluation of the organizational-level determinants of participation in workplace health promotion programs, Lier and colleagues found that organizational size was negatively correlated with participation (31). They also found that organizational support was a significant predictor of participation (31). This is in alignment with the finding of Lau and Faulkner (10) in leadership support being a key factor in the success of the UPnGO program. As such, future interventions should focus on increasing the organizational support of PA in the workplace and providing permission for employees to participate in PA during the workday especially in larger companies.

Overall, commercializing the program resulted in changes to the “active ingredients” that drove the success of the 6-week pilot program (10): senior management role modeling, individual self-monitoring of steps through provision of subsidized devices for tracking, and reduced visibility of the implementation team in the workplace. We hypothesize that the loss of these “active ingredients,” in conjunction with the extended length of the program to 1 year, were key barriers to the sustainability of the program.

## Limitations

There are several limitations to this study. The primary limitations being the limited recruitment and declining engagement of participants (~30% of eligible employees), and the lack of recruitment of the target group, physically inactive individuals. This led to a small and likely biased sample included in the effectiveness analysis. Additionally, as a result of commercialization, the companies were charged for use of the program which may have contributed to selection bias in terms of participating companies. The use of personal tracking devices to derive device-recorded data resulted in the grouping of step data from a variety of different tracking devices, each with their own internal logic, reliability, and validity. Also, the use of self-reported physical activity and sitting time is also subject to recall bias. Finally, there was no follow-up with the companies following the 12 months of the program to qualitatively explore perceptions of the program.

## Conclusions

The commercialization of UPnGO was an ambitious goal in moving from a successful pilot program to a commercially available mHealth PA intervention. Although ultimately unsuccessful some key lessons can be generated from the attempt. The workplace remains an important environment for PA interventions, but effective PA programs are difficult to implement and sustain. Commercialization adds an additional level of complexity to the scaling-up process of PA programs and may influence participant expectations. While interest in mHealth research related to PA and sedentary behavior has grown rapidly, sustaining sufficient engagement necessary for long-term behavior change remains elusive. More research is needed in understanding how to sustain engagement and in identifying the optimal length of PA mHealth interventions for the workplace.

## Data Availability Statement

The data analyzed in this study is subject to the following licenses/restrictions: This study involved the secondary data analysis of the process and outcome evaluation of the UPnGO with ParticipACTION evaluation. The data is owned by ParticipACTION and was provided to the research team as third party evaluators. Requests to access these datasets should be directed to guy.faulkner@ubc.ca.

## Ethics Statement

The studies involving human participants were reviewed and approved by the University of British Columbia Behavioral Research Ethics Board. Written informed consent for participation was not required for this study in accordance with the national legislation and the institutional requirements.

## Author Contributions

KD and GF conceived of the manuscript and wrote the manuscript. KD, EL, and GF developed the manuscript idea. KD and LY collected the data. KD analyzed the data. All authors revised and approved of the final manuscript.

## Funding

UPnGO with ParticipACTION was supported by a multi-sectoral partnership grant from the Public Health Agency of Canada, ParticipACTION, Public Inc., the British Columbia Ministry of Health, and the Ontario Trillium Foundation and AstraZeneca. KD and LY received funding for their work through Mitacs Accelerate Awards in collaboration with ParticipACTION.

## Conflict of Interest

KD and LY received funding through Mitacs Accelerate Awards in partnership with ParticipACTION. GF was a member of the ParticipACTION Research Advisory Group. The remaining author declares that the research was conducted in the absence of any commercial or financial relationships that could be construed as a potential conflict of interest.

## Publisher's Note

All claims expressed in this article are solely those of the authors and do not necessarily represent those of their affiliated organizations, or those of the publisher, the editors and the reviewers. Any product that may be evaluated in this article, or claim that may be made by its manufacturer, is not guaranteed or endorsed by the publisher.
